# Functional consequences of C-terminal mutations in *RUNX2*

**DOI:** 10.1038/s41598-023-39293-1

**Published:** 2023-07-27

**Authors:** Sermporn Thaweesapphithak, Thanakorn Theerapanon, Khanti Rattanapornsompong, Narin Intarak, Pimsiri Kanpittaya, Vorapat Trachoo, Thantrira Porntaveetus, Vorasuk Shotelersuk

**Affiliations:** 1 Center of Excellence in Genomics and Precision Dentistry, Department of Physiology, Faculty of Dentistry, Chulalongkorn University, Bangkok, 10330 Thailand; 2 Graduate Program in Oral Biology, Faculty of Dentistry, Chulalongkorn University, Bangkok, Thailand; 3 Department of Orthodontics, Faculty of Dentistry, Chulalongkorn University, Bangkok, Thailand; 4 Department of Oral and Maxillofacial Surgery, Faculty of Dentistry, Chulalongkorn University, Bangkok, Thailand; 5 Graduate Program in Geriatric and Special Patients Care, Faculty of Dentistry, Chulalongkorn University, Bangkok, Thailand; 6 Center of Excellence for Medical Genomics, Department of Pediatrics, Faculty of Medicine, Chulalongkorn University, Bangkok, Thailand; 7 Excellence Center for Genomics and Precision Medicine, King Chulalongkorn Memorial Hospital, the Thai Red Cross Society, Bangkok, Thailand

**Keywords:** Dental diseases, Oral diseases, Genetics, Stem cells

## Abstract

Cleidocranial dysplasia (CCD) is a genetic disorder caused by mutations in the *RUNX2* gene, affecting bone and teeth development. Previous studies focused on mutations in the *RUNX2* RHD domain, with limited investigation of mutations in the C-terminal domain. This study aimed to investigate the functional consequences of C-terminal mutations in *RUNX2*. Eight mutations were analyzed, and their effects on transactivation activity, protein expression, subcellular localization, and osteogenic potential were studied. Truncating mutations in the PST region and a missense mutation in the NMTS region resulted in increased transactivation activity, while missense mutations in the PST showed activity comparable to the control. Truncating mutations produced truncated proteins, while missense mutations produced normal-sized proteins. Mutant proteins were mislocalized, with six mutant proteins detected in both the nucleus and cytoplasm. CCD patient bone cells exhibited mislocalization of RUNX2, similar to the generated mutant. Mislocalization of RUNX2 and reduced expression of downstream genes were observed in MSCs from a CCD patient with the p.Ser247Valfs*3 mutation, leading to compromised osteogenic potential. This study provides insight into the functional consequences of C-terminal mutations in *RUNX2*, including reduced expression, mislocalization, and aberrant transactivation of downstream genes, contributing to the compromised osteogenic potential observed in CCD.

## Introduction

Runt-related transcription factor 2 (*RUNX2*, NM_001024630.4) is a key regulator of bone formation by controlling the differentiation of osteoblasts and chondrocytes. Heterozygous loss-of-function mutations in *RUNX2* lead to cleidocranial dysplasia (CCD, MIM #119600), while gain-of-function mutations result in metaphyseal dysplasia with maxillary hypoplasia with or without brachydactyly (MDMHB, MIM #156510) and nonsyndromic midline craniosynostosis^1–3^.

The phenotypic penetrance of *RUNX2* variants in CCD displays heterogeneity and variable expression, including among family members. Establishing a clear relationship between genotype and phenotype in CCD has been challenging^4,5^. Patients with mutations in either the RHD (S118R, F121C, T205G, R225W, and R225Q) or the C-terminal region (G363V and G428Afs*56) exhibit classic CCD symptoms, including clavicle defects, delayed fontanelle closure, midface hypoplasia, delayed tooth eruption, and supernumerary teeth, however, expressivity can vary greatly^6^. Intrafamilial expressivity variation is also frequently observed among patients with the same mutations^4,5^. Consistent with other reports, our previous study, which included patients with mutations in both the RHD and the C-terminus, also noted variable disease severity^7^. The study revealed a range of dental phenotypes, including 19–30 unerupted teeth and 5–8 supernumerary teeth, as well as variations in height, the degree of maxillary hypoplasia, and the severity of clavicular hypoplasia. Moreover, specific manifestations, such as brachymetatarsia and partial pseudoepiphysis of the proximal metacarpal bone were observed^7^. These findings collectively emphasize the heterogeneity in the phenotypic presentation of CCD patients, highlighting the complex nature of genotype–phenotype correlations in CCD. Furthermore, the CCD phenotype might be influenced by the interactions of *RUNX2* with other genes.

RUNX2 is a multidomain protein that consists of different functional regions. The N-terminal portion of RUNX2 contains two important domains: the glutamine-alanine (QA) repeated region and the runt homologous domain (RHD). The QA region plays a role in the transactivation activity of *RUNX2*. Deleting the QA region has been shown to lead to a reduction in the transactivation activity of RUNX2^8^. The RHD serves as the DNA-binding region of RUNX2, enabling it to interact with specific genes involved in various developmental processes. For instance, RUNX2 interacts with genes, such as osteocalcin (*OCN*), osterix (*OSX*), and matrix metalloproteinase 13 (*MMP13*), which are critical for osteogenesis and inflammation. Additionally, the RHD interacts with a coactivator called core-binding factor subunit beta (CBFβ). This interaction with CBFβ is crucial for stabilizing RUNX2 during skeletogenesis, ensuring its proper functioning and activity in skeletal development^9^. A nine amino acid sequence adjacent to the RHD is the nuclear localization signal (NLS) region that is important for RUNX2 nuclear localization. Mutations in the NLS results in a mislocalization of RUNX2 in the cytoplasm. The C-terminus of RUNX2 contains the proline-serine-threonine-rich (PST), nuclear matrix targeting signal (NMTS), and VWRPY sequence regions. The PST region plays a crucial role in interacting with co-activators and co-repressors, which are involved in the regulation of downstream genes^10^. Within the PST region, the NMTS is responsible for the proper nuclear localization of RUNX2. Additionally, the last five amino acids of the PST region, known as VWRPY, are conserved among RUNT families and serve as a transcriptional repression domain. They act as an interacting domain for co-regulatory proteins involved in cell signaling pathways, ultimately activating RUNX2 target genes^11^. Furthermore, studies have shown that deleting the C-terminus of RUNX2 results in complete bone loss, similar to the phenotype observed in RUNX2 null mutants. This finding highlights the crucial role of the C-terminus in the functioning of RUNX2^9,12^.

*RUNX2* regulates downstream target genes by direct binding through the osteoblast-specific cis-acting element (OSE2), which is found in the promoters of several bone-specific genes, including *OCN*, osteopontin (*OPN*), bone sialoprotein (*BSP*), and collagen, type I, alpha-1 (*COL1A1*)^10^. *RUNX2* also regulates craniosynostosis-related genes, including skeletal tissue-enriched gene Pannexin3 (*PANX3*), skeletal tissue enriched gene matrix metalloproteinase 9 (*MMP9*), *MMP13*, and neural epidermal growth factor-like protein 1 (*NELL1*)^13,14^. This highlights the broad range of functions attributed to RUNX2 in various biological contexts.

The identification and characterization of novel variants are crucial for both research and clinical applications, particularly in disease prognosis and genetic counseling. Although many mutations in the *RUNX2* gene have been identified in patients with CCD, most of these mutations occur in the RHD, which has been extensively studied for its functional consequences^6^. Conversely, mutations in the C-terminus of RUNX2 have received less validation, impeding our understanding of the C-terminus’ role. The aims of this study were (1) to investigate the functional consequences of C-terminal mutations, including missense and truncating variants located in the PST or NMTS region, (2) to explore the subcellular localization and osteogenic potential of alveolar bone mesenchymal stem cells (aBMSC) derived from a CCD patient with the p.Ser247Valfs*3 mutation, and (3) to review and summarize the existing functional studies on C-terminal mutations in RUNX2.

## Results

**Figure 1:**
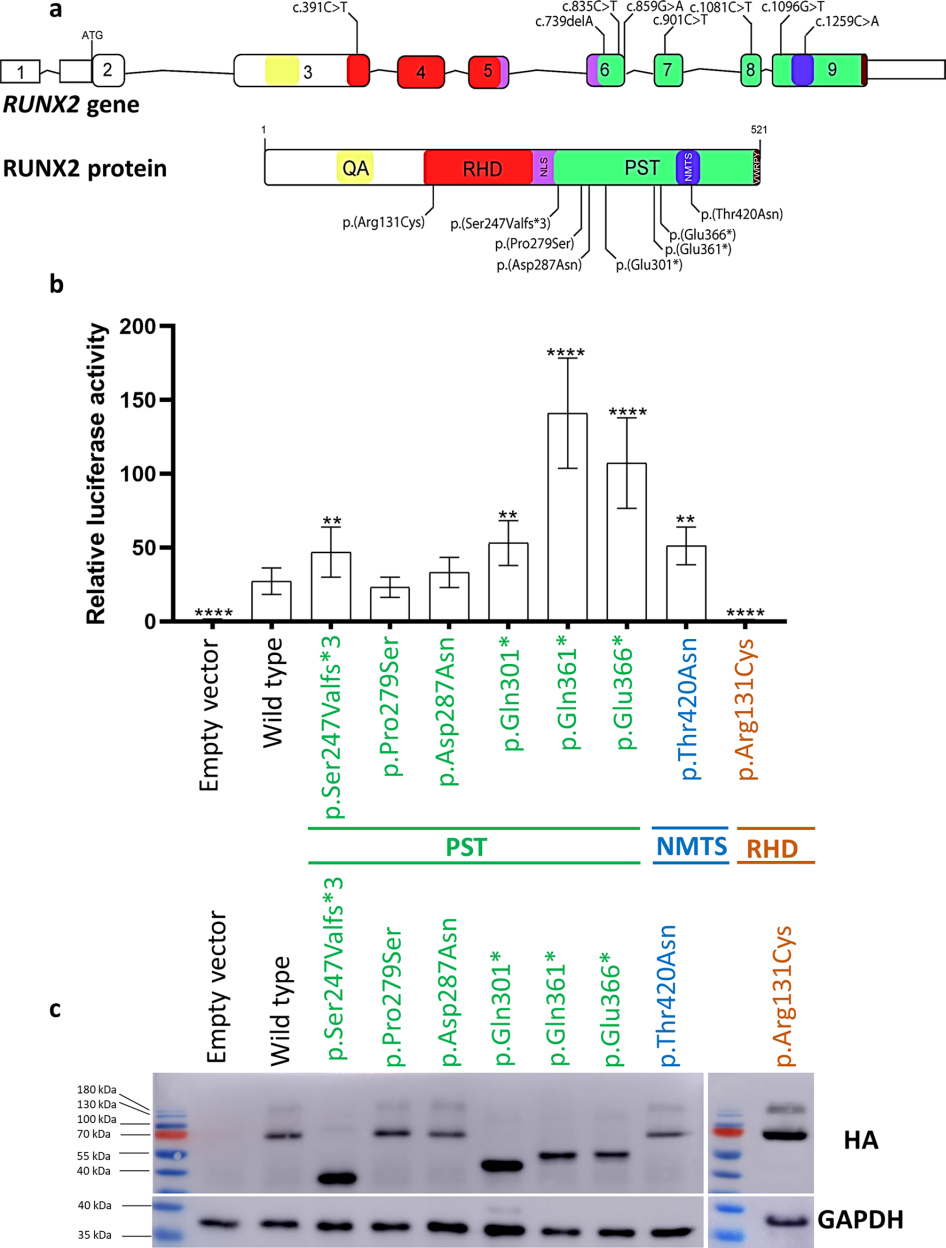
Functional analysis of *RUNX2* mutations included in this study. (**a**) Schematic diagrams demonstrating the variants included in this study: c.391C > T (p.Arg131cys) in RHD, c.739delA (p.Ser247Valfs*3), c.835C > T (p.Pro279Ser), c.859G > A (p.Asp287Asn), c.901C > T (p.Gln301*), c.1081C > T (p.Gln361*), c.1096G > T (p.Glu366*) in PST, and c.1259C > A (p.Thr420Asn) in NMTS. (**b**) Transactivation of the pOSE2-6Luc reporter by *RUNX2* mutations compared with WT (WT-HA-RUNX2-pcDNA3.1[ +]) and empty vector. The results are presented as relative light units (RLUs) of tested variants normalized to Renilla. (**c**) Western blot of mutant RUNX2 and WT. GAPDH was used for the loading control. Full-length blots are presented in Fig. S3-6. The negative control was a lysate of an empty vector (pcDNA3.1[ +]) transfected into HEK293 cells. A significant difference from wild type: **P ≤ 0.05; ****P ≤ 0.0005. *RHD* runt homology domain, *PST* proline/serine/threonine-rich, *NMTS* nuclear matrix-targeting signal.

The present study investigated the functional consequences of seven C-terminal mutations and one previously tested mutation in the RHD of RUNX2 (used as a control) (Fig. 1a). We conducted experiments using mutation-transfected cells and examined primary alveolar bone cells from a CCD patient to gain insights into the effects of these mutations.

A dual luciferase assay revealed that the truncating variants in the PST region [p.Ser247Valfs*3, p.Gln301*, p.Gln361*, p.Glu366*] and the missense variant in the NMTS [p.Thr420Asn] exhibited significantly increased transcriptional activities. In contrast, the missense variants in the PST region [p.Pro279Ser and p.Asp287Asn] showed comparable activities to the wild-type (WT), indicating that these PST-missense mutations are unlikely to disrupt the function of OCN. To validate the luciferase assay, we conducted further investigations on the previously tested variant p.Arg131Cys in the RHD. Our findings confirmed that this variant exhibited decreased transactivation of the OCN promoter, consistent with a previous study^15^ (Fig. 1b).

To assess the impact of the mutations on protein expression, we conducted western blotting analysis. Our results revealed that all missense variants [p.Pro279Ser, p.Asp287Asn, p.Thr420Asn, p.Arg131Cys] exhibited full-length RUNX2 protein expression similar to the WT. In contrast, all frameshift and nonsense variants [p.Ser247Valfs*3, p.Gln301*, p.Gln361*, p.Glu366*] showed smaller protein bands, compared with the WT. These findings indicate that the protein products of missense mutations were unaffected in size, while those resulting from nonsense and frameshift mutations were truncated (Fig. 1c).

**Figure 2:**
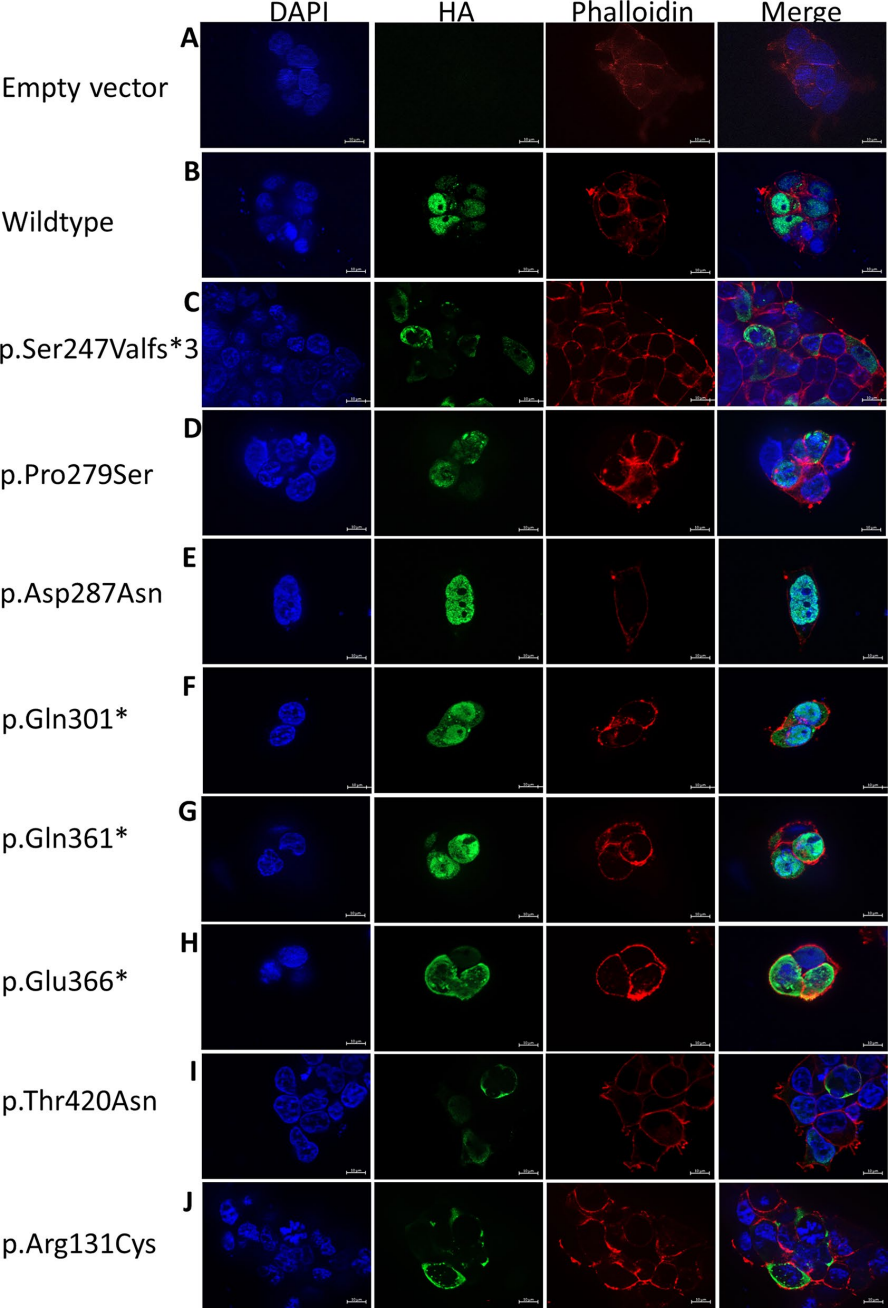
Subcellular localization of mutant RUNX2 in HEK293 cells. (**A**) Empty vector (pcDNA3.1[ +]). (**B**) wildtype. (**C**) p.Ser247Valfs*3. (**D**) p.Pro279Ser. (**E**) p.Asp287Asn. (**F**) p.Gln301*. (**G**) p.Gln361*. (**H**) p.Glu366*. (**I**) p.Thr420Asn. (**J**) p.Arg131Cys. In this figure, Z-stack imaging was performed to capture the focal plane of the *RUNX2*-transfected cells.

Immunolocalization was employed to examine the subcellular localization of the RUNX2 variants in the mutant cells. The nonsense and frameshift variants in the PST region [p.Ser247Valfs*3, p.Gln301*, p.Gln361*, p.Glu366*] and the missense variant in the NMTS [p.Thr420Asn] were detected in both the nucleus and cytoplasm. In contrast, the missense variant in the PST region, p.Asp287Asn, previously reported in CCD patients^16,17^, exhibited nuclear localization similar to the WT, while the p.Pro279Ser variant, not found in any CCD patients, was observed in both the cytoplasm and nucleus. These findings indicate differential localization patterns of CCD-causing mutations in the C-terminus, with all C-terminal mutations, except the PST missense variant, impairing the nuclear accumulation of RUNX2. Furthermore, the p.Arg131Cys variant in the RHD region was localized exclusively in the cytoplasm, consistent with previous research^15^ (Fig. 2).

**Figure 3:**
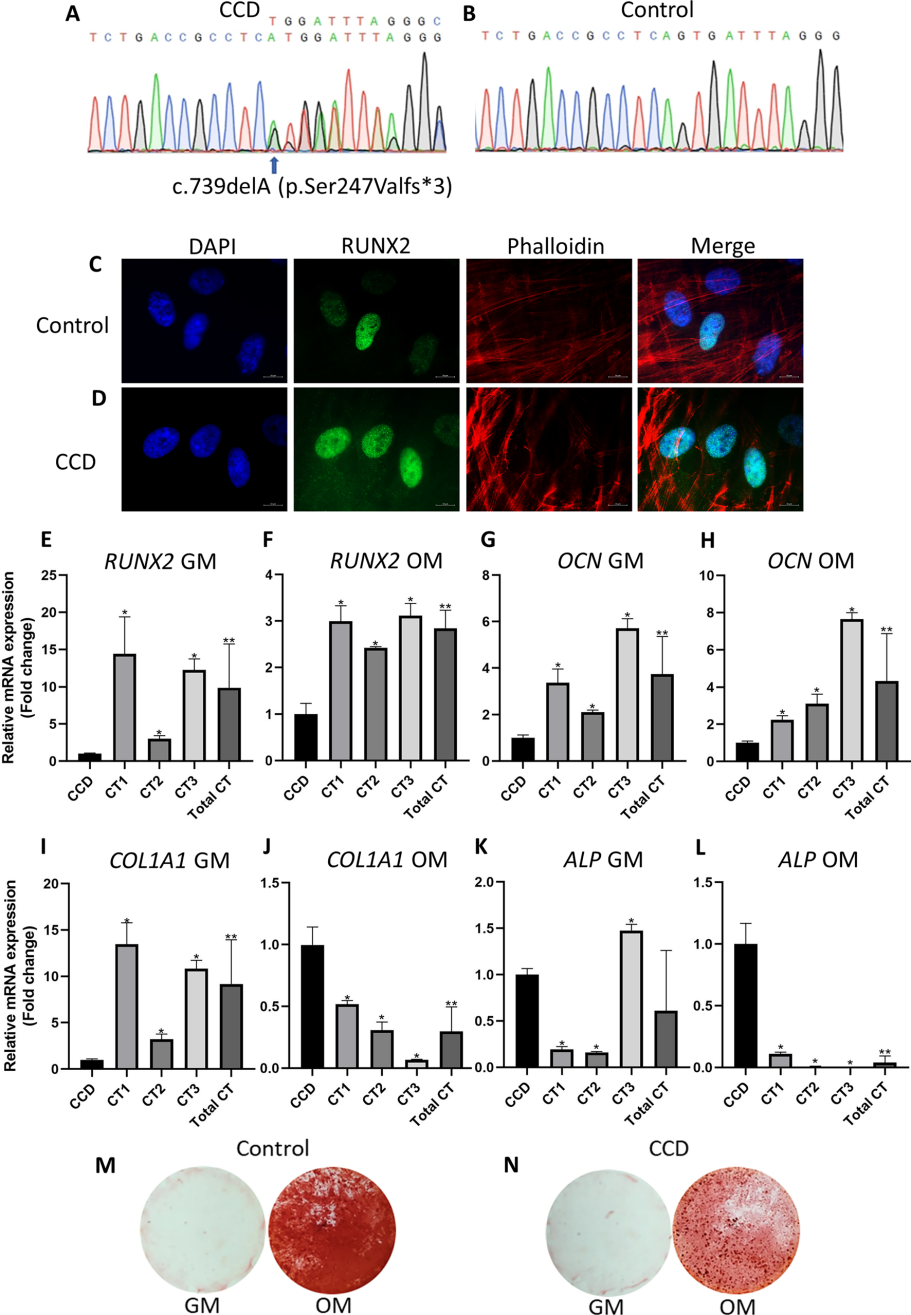
Characterisitcs of aBMSCs with p.Ser247Valfs*3 mutation. (**A**,**B**) Chromatograms illustrate the heterozygous missense mutation, c.739delA (p.Ser247Valfs*3) in the *RUNX2* gene (NM_001024630.4) in the CCD cells obtained from a patient affected with CCD. The mutation was absent in control cells. (**C**,**D**) Subcellular localization of CCD and control cells. (**E**-**L**) The expression of *RUNX2*, *OCN*, *COL1A1*, and *ALP* mRNA in CCD and control cells cultured in general medium (GM) or osteogenic medium (OM). (**M**,**N**) Alizarin red S staining of CCD and control cells culture in OM.

To investigate the molecular mechanisms underlying the C-terminal variant in human pathogenesis, we examined aBMSCs obtained from a CCD patient with the c.739delA (p.Ser247Valfs*3) mutation in the PST region (referred to as CCD cells). Three control aBMSC samples were also obtained from healthy individuals for comparison. Sanger sequencing verified the presence of the p.Ser247Valfs*3 mutation in the CCD cells (Fig. 3A,B). Furthermore, both the CCD cells and control samples were assessed for mesenchymal stem cell marker expression, which confirmed their mesenchymal stem cell identity based on positive expression of CD44, CD73, CD90, and CD105, and negative expression of CD45 (Fig. S2). Immunolocalization analysis was performed to evaluate the subcellular localization of RUNX2 in CCD cells compared with cells transfected with the p.Ser247Valfs3 mutation. The control cells displayed nuclear localization of RUNX2, while both the CCD cells and p.Ser247Valfs*3 transfected cells exhibited RUNX2 localization in both the nucleus and cytoplasm (Figs. 2C, 3C,D). These observations confirm that the p.Ser247Valfs*3 mutation disrupts the nuclear accumulation of RUNX2.

To assess the impact of the p.Ser247Valfs*3 mutation on osteogenic differentiation in CCD cells, we evaluated the expression of bone-related genes and performed alizarin red S staining. Real-time PCR analysis demonstrated a significant decrease in the expression of *RUNX2* and *OCN* mRNA in CCD cells compared with controls, in both general medium (GM) and osteogenic medium (OM) (Fig. 3E–H). Furthermore, in GM, CCD cells exhibited a significant downregulation of *COL1A1*, which was subsequently significantly upregulated in response to osteogenic induction, unlike in controls (Fig. 3I,J). Similarly, in OM, CCD cells showed a significantly higher expression of *ALP* compared with controls (Fig. 3K,L). These findings indicate that CCD cells display altered expression of bone-related genes under both normal and osteogenic conditions. Moreover, the levels of *RUNX2* and *OCN* expression are likely compromised, including when CCD cells are subjected to OM. Additionally, alizarin red S staining revealed reduced calcium deposition in CCD cells compared with controls, suggesting impaired mineral deposition ability (Fig. 3M,N). Taken together, our results suggest that the p.Ser247Valfs*3 mutation may impact the osteogenic differentiation of aBMSCs.

We conducted a comprehensive review of the published functional studies on the C-terminal mutations of RUNX2, as summarized in Table 1^7,16–37^. Regarding subcellular localization, previous studies reported that mutations in the PST region were observed either exclusively in the nucleus or in both the nucleus and cytoplasm. In our study, all PST mutations were detected in both cellular compartments. For the NMTS region, a previously reported p.Arg391* mutation was found exclusively in the nucleus, while the p.Thr420Asn mutation in our study was observed in both the nucleus and cytoplasm. In terms of protein size, our findings were consistent with the type of mutation. Truncating mutations in *RUNX2* resulted in a smaller protein size, while missense mutations showed a normal protein size. The luciferase assay results showed a discrepancy compared with previously reported C-terminal mutations, which showed reduced transactivation of the *OCN* promoter. In contrast, we observed that truncating mutations in PST and the missense mutation in NMTS exhibited increased transactivation activities, while missense mutations in PST showed activities comparable to the WT. Furthermore, we compared the expression of bone-related markers and the osteogenic potential of *RUNX2* mutant cells between our study and previous studies. Although the results varied, likely due to different mutations and cell types used in the studies, all mutant cells or tissues exhibited compromised alizarin red staining after osteogenic induction and showed altered expression of bone-related genes. These findings indicate that mutations in the C-terminus of RUNX2 interfere with the osteogenic ability of cells.

**Table 1:** A summary of the functional studies of the C-terminal mutations in *RUNX2*.

Genetic variant	Amino acid change	Cell type	Localization	Transactivation	Protein expression	Gene expression and osteogenic differentiation	Articles reporting the variants in CCD patients	Functional studies
PST region								
c.739delA	p.Ser247Valfs*3	HEK-293T	Nucleus and cytoplasm	High	Truncated	NA	^7^	This study
c.739delA	p.Ser247Valfs*3	aBMSC	Nucleus and cytoplasm	NA	NA	↓ *RUNX2, OCN, COL1A1;* ~ *ALP* (GM) ↓ *RUNX2, OCN;* ↑ *COL1A1, ALP* ↓ ARS staining	^7^	This study
c.774_779del AGTAGGinsGA	p.Val259Metfs*11^π^	HEK-293T	Nucleus	Low	Truncated	NA	^18^	^18^
c.835C > T	p.Pro279Ser	HEK-293T	Nucleus and cytoplasm	Normal	Normal	NA	None	This study
c.838C > T	p.Gln280*	NIH-3T3	Nucleus and cytoplasm	Half	NA	NA	^19^^†^	^19^^†^
c.859G > A	p.Asp287Asn	HEK-293T	Nucleus	Normal	Normal	NA	^16,17^	This study
c.887dup	p.Trp297Valfs*4^θ^	CHO	Nucleus and cytoplasm	NA	NA	NA	^20^	^20^
c.897 T > G	p.Tyr299*	Fibroblasts	NA	NA	Truncated	↓ *SPARC, P53, and PTEN*	^21^	^21^
c.901C > T	p.Gln301*	HEK-293T	Nucleus and cytoplasm	High	Truncated	NA	^7^	This study
c.1019del	p.Ser340*	Fibroblasts, cMSCs	NA	NA	Truncated	↓ *SPARC, P53, and PTEN*	^21^	^21^
c.1081C > T	p.Gln361*	HEK-293T	Nucleus and cytoplasm	High	Truncated	NA	^7^	This study
c.1085C > T	p.Ala362Val^#^	NIH-3T3	Nucleus and cytoplasm	Half	NA	NA	^19^^†^	^19^^†^
c.1096G > T	p.Glu366*	HEK-293T	Nucleus and cytoplasm	High	Truncated	NA	^22^	This study
c.1096G > T	p.Glu366*	DPCs	Nucleus and cytoplasm	NA	Truncated	NA	^22^	^22^
c.1111dup	p.Ser371Phefs*14	HEK-293T	Nucleus and cytoplasm	Low	Truncated	NA	^23^	^23^
c.1119dup	p.Arg374Glnfs*11	BMMSCs, DPSCs	NA	NA	Truncated	↑ *RUNX2*, *ALP*, ~ *OCN* (GM) ↓ *RUNX2, ALP, OCN* (OM) ↓ ARS staining	^24^	^24^
NMTS region								
c.1171C > T	p.Arg391*	NIH 3T3, COS7	NA	Low	Truncated	NA	^25–35^^†^	^35^^†^
c.1199dup	p.Tyr400*	HEK-293T	Nucleus	Low	Truncated	NA	^18^	^18^
c.1228dup	p.Leu410Profs*80	Growth plate RNA	NA	NA	NA	↓ *RUNX2*, *COL10A1*, *MMP13*, *VEGF* ↑ *RUNX3*	^36^	^36^
c.1259C > A	p.Thr420Asn	HEK-293T	Nucleus and cytoplasm	High	Normal	NA	^16^	This study
c.1384G > T	p.Gly462*	C2C12	NA	Low	Truncated	↓ *OCN, ALP, COL1A1*, *MMP13, BSP*, *OPN;* ↓ ARS staining	^33^	^33^
VWRPY region								
c.1550del	p.Trp518Glyfs*60	HEK-293T	Nucleus	Low	Elongated	NA	^37^	^37^

*aBMSC* alveolar bone mesenchymal stem cells, *ARS* alizarin red S, *cMSCs* circulating mesenchymal stem cells, *DPCs* dental pulp cells, *GM* general media, *NA* not applicable, *OM* osteogenic media.

↓, ↑,  ~ , decreased, increased, comparable level compared with controls, respectively.

^#^predicted splicing (https://varseak.bio/index.php), ^θ^p.Val259Metfs*11 (p.258 fs reported in an original article), ^π^p.Trp297Valfs*4 (p.296 fs reported in an original article), ^†^NM_001024630.4 (NM_001369405.1 reported in an original article).

## Discussion

The p.Arg131Cys in RHD was previously tested in vitro and used as a positive control to validate our experiments. The RHD is highly conserved and responsible for DNA-binding and heterodimerization with CBFβ^6,21^. The mutation in RHD resulted in a protein that was unable to bind DNA or co-factor CBFβ^38^. Our results showed that the p.Arg131Cys completely abolished the transactivation activity on the OCN promoter and was mislocalized in the cytoplasm, while the protein size was normal. These findings are consistent with a previous study and validate our results^39^. Interestingly, three different substitutions at the p.Arg131 position have been reported. In contrast to p.Arg131Cys, which was localized in the cytoplasm, p.Arg131Gly was detected in the nucleus similar to WT, but exhibited abrogated transactivation^40,41^, indicating that the variants in the same amino acid position could have different pathogenicity.

The PST region plays a critical role in the transcriptional regulation mediated by RUNX2, as well as its interaction with other transcription factors, co-activators, and co-repressors^42^. Previous studies have demonstrated that *RUNX2* mutations lead to a substantial reduction in transactivation activity of the osteocalcin promoter^42^. However, we observed that all truncating variants in the PST region, as well as a missense variant in NMTS, exhibited a significant increase in transactivation activity. The PST region is known to interact with various transcriptional coactivators and corepressors. For instance, it can interact with the coactivator p300, which facilitates the activation of target genes. Additionally, it can also interact with corepressors, such as Histone deacetylase 6 (HDAC6) and Groucho/TLE, leading to the repression of target genes^43,44^. These interactions within the PST region contribute to the dynamic regulation of target gene expression. Previous studies have demonstrated that deleting the last 154 amino acids of RUNX2, which encompasses a portion of the PST region and the entire transcriptional repressor VWRPY sequence, leads to a significant increase in transcriptional activities^42^. Furthermore, alterations in the expression levels of RUNX2, either through gain or loss of function, have been shown to impact osteoblastic differentiation. RUNX2 haploinsufficiency has been associated with CCD, characterized by underdevelopment of the clavicles, dental abnormalities, and incomplete fusion of cranial sutures. The severity of the CCD symptoms varies depending on the specific nature of the *RUNX2* mutations. In contrast, excessive dosage of RUNX2 leads to craniosynostosis, a condition characterized by premature fusion of cranial sutures^1^. These findings emphasize the critical role of RUNX2 in regulating osteoblastic differentiation and craniofacial development. Both loss and gain of RUNX2 function can have profound effects on skeletal and cranial development, underscoring the precise regulation required for proper bone formation and suture fusion.

The precise molecular mechanism underlying how increased transactivation activity contributes to CCD remains unclear. However, the association may be explained by several possibilities. One possibility is that the mutant form of RUNX2 lacks the repressor function normally exerted by the wild-type protein. This loss of repressor activity could result in the aberrant activation of downstream targets or partners of RUNX2. Another possibility is that the structural modifications caused by the mutation in the C-terminal region of RUNX2 affect its secondary or tertiary structure. These structural alterations might disrupt the binding of RUNX2 to DNA sequences within its target genes, as well as its interactions with other transcriptional cofactors, transcription factors, or repressors. Consequently, the normal regulatory functions of RUNX2 in controlling gene expression may be perturbed. It is important to note that although the transactivation assay in the present study focused on the osteocalcin promoter, RUNX2 is involved in the regulation of numerous genes associated with various pathways^9^. Therefore, relying solely on the OCN promoter in transactivation studies might not fully capture the complete biological function of RUNX2. Additional functional investigations are necessary to investigate the pathogenesis of C-terminal mutations in RUNX2 more deeply and elucidate their precise impact on cellular processes.

In the PST region, we observed that all truncating variants [p.Ser247Valfs*3, p.Gln301*, p.Gln361*, p.Glu366*] produced truncated proteins, suggesting that the mutant mRNA was likely to escape the nonsense-mediated mRNA decay^44^. In addition, all variants showed impaired localization and were found in both the nucleus and cytoplasm, consistent with previous studies^18,45^. The heterozygous p.Glu366* was previously reported in a CCD patient, and the p.Glu366*-dental pulp cells showed lower proliferation and differentiation than controls^42^. Furthermore, the p.Glu366* exhibited a truncated protein lacking 155 amino acids, and the RUNX2 localization in the patient’s primary cells were found in both the nucleus and cytoplasm^43,44^. Our findings regarding the p.Glu366*-transfected cells align with the observed effects of truncating variants in the PST region. The presence of a truncated protein, impaired subcellular localization of RUNX2, and disturbed transactivation collectively suggest a potential pathogenic mechanism for these truncating variants in the PST region.

Contrary to the truncating mutations, we observed that the missense variants in PST exhibited protein size and transactivation activity similar to WT protein. The p.Asp287Asn was localized in the nucleus, while the p.Pro279Ser was detected in both nucleus and cytoplasm. Thus, the p.Pro279Ser might change the protein structure and affect protein subcellular localization. The above findings indicate that the truncating and the missense variants in PST have different functional effects.

NMTS, which resides in the PST region, participates in RUNX2 subnuclear localization and has binding affinity to other proteins^43,46^. Three truncating variants in NMTS [p.Arg391*, p.Tyr400*, p.Gly462*] were previously shown to produce smaller proteins and decreased transactivation, and the p.Tyr400* has also been reported to be localized in the nucleus similar to WT^28,47,48^. Our study, for the first time, demonstrated the functional effects of the missense variant in NMTS. The p.Thr420Asn exhibited significantly increased transcriptional activity and impaired subcellular localization was observed in both the nucleus and the cytoplasm, while it produced a protein of normal size. These findings suggest different pathogenic mechanisms between the missense and truncating mutations within the NMTS region.

Regarding gene expression, the bone marrow and dental pulp mesenchymal stem cells from a patient carrying the *RUNX2* p.Arg374Glnfs*11 (p.Q374fsX384 in original article) mutation exhibited higher expression of *RUNX2* and *ALP* compared with controls. However, the expression of *RUNX2, ALP,* and *OCN,* as well as mineral deposition, were decreased after osteogenic induction^24^. Growth plate RNA from a patient with the p.Leu410Profs*80 mutation exhibited a decrease in *RUNX2, COL10A1, MMP13,* and *VEGF* expression, but an increased *RUNX3* expression^36^. The C2C12 cells transfected with the p.Gly462* variant demonstrated reduced *OCN, ALP, COL1A1, MMP13, BSP,* and *OPN* expression and the cells, after osteogenic induction, showed reduced mineral deposition^33^. These data show that different cell types carrying *RUNX2* mutations have varying osteogenic differentiation potential. Here, we examined alveolar bone cells, which served as a representative model for the bone defects commonly found in patients with CCD, and demonstrated that aBMSCs from a patient with the p.Ser247Valfs*3 exhibited reduced *RUNX2, OCN,* and *COL1A1* expression compared with controls, However, the expression of *ALP* was comparable between CCD cells and the control cells. After osteogenic induction, *RUNX2* and *OCN* expression in CCD cells remained lower than controls. Interestingly, *COL1A1* and *ALP* were upregulated in CCD cells compared with controls. These findings might suggest that *RUNX2* and *OCN* expression in the *RUNX2*-mutated CCD cells were compromised, while *COL1A1* and *ALP* can be induced upon osteogenic manipulation. Furthermore, we examined the localization of RUNX2 in the p.Ser247Valfs*3 aBMSCs and found that RUNX2 was located in both the nucleus and cytoplasm, similar to the localization pattern observed in p.Ser247Valfs*3-transfected HEK-293 cells. Altogether, our findings indicate that the p.Ser247Valfs*3 leads to the production of a truncated protein, which in turn affects its subcellular localization, transactivation, and expression of genes related to bone development.

## Conclusion

This study reported altered functional consequences of the cells transfected with different variants located in the C-terminus of RUNX2, including both the PST and NMTS regions, and altered osteogenic potential of primary aBMSCs carrying the p.Ser247Valfs*3 in PST. We demonstrated that the functional effects of *RUNX2* mutations are influenced by the specific location and type of mutation (truncating or missense), as well as the cell types used in the study. Our findings enrich the evidence for the functional consequences of C-terminal mutations of *RUNX2* that are responsible for CCD.

## Materials and method

### Ethics approval and consent to participate

The project entitled “study of mutation and pathomechanism of *RUNX2* in cleidocranial dysplasia” was approved by the Human Research Ethics Committee of the Faculty of Dentistry, Chulalongkorn University, Thailand (Approval No. 036/2021, Date of approval 9th July 2021). All experiments were carried out in accordance with the Helsinki Declaration and relevant guidelines and regulations. Written informed consent was obtained from each participant.

### Selection of C-terminal mutations in RUNX2

For the functional analyses, we selected seven *RUNX2* mutations that have not been functionally evaluated (Fig. 1, Table 1) consisting of nonsense, frameshift, and missense mutations in PST and NMTS regions. Three variants in PST were reported in our previous study [c.739delA (p.Ser247Valfs*3), c.901C > T (p.Gln301*), c.1081C > T (p.Gln361*)]^7^, two variants [c.859G > A (p.Asp287Asn) in PST, c.1259C > A (p.Thr420Asn) in NMTS] by Ott et al*.*^16^, one variant, c.1096G > T (p.Glu366*), by Xuan et al.^22^, and one variant, c.835C > T (p.Pro279Ser), has not been identified in patients with CCD. We also used the c.391C > T (p.Arg131Cys) in RHD that was previously tested as a control^15^.

### Amino acid alignment

To determine the conservation of amino acids, the substituted amino acids of the selected missense mutations comprising p.Arg131, Pro279, Asp287, and Thr420 were aligned among several species: Homo sapiens (NP_001019801.3), Mus musculus (XP_006523603.1), Rattus norvegicus (XP_006244611.1), Xenopus tropicalis (NP_001128588.1), Danio rerio (NP_998023.1), Drosophila melanogaster (NP_001285501.1), and Caenorhabditis elegans (NP_491679.1) using Marrvel^49^ (Fig. S1).

### Plasmid constructs

The empty vector (pcDNA3.1[ +]), wild type (WT) clone tagged with hemagglutinin (HA) (WT-HA-RUNX2-pcDNA3.1[ +]), p.Arg131Cys (R131C-HA-RUNX2-pcDNA3.1[ +]), and pOSE2-6Luc reporter plasmid were kindly provided by Dr. Ewa Hordyjewska-Kowalczyk, Department of Biomedical Sciences, Laboratory of Molecular Genetics, Medical University of Lublin, Poland^15^. The WT-HA-RUNX2-pcDNA3.1[ +] was used as a template. The p.Arg131Cys was used as a positive control. Mutagenesis was performed using the Q5® site-directed mutagenesis kit (New England Biolabs, MA, USA) with the primers shown in Table S1, The PCR products were digested with the restriction enzymes NotI-HF® and XbaI (New England Biolabs) and ligated into WT-HA-RUNX2-pcDNA3.1[ +] by T4 DNA Ligase (New England Biolabs). The mutagenesis products were confirmed by Sanger sequencing using the primers presented in Table S2.

### Cell culture

Human embryonic kidney 293 (HEK293, CRL-1573) cells obtained from the American Type Culture Collection (ATCC, VA, USA) were used for functional studies. The primary alveolar bone mesenchymal stem cells (aBMSCs) obtained from the patient harboring the c.739delA, (p.Ser247Valfs*3) mutation in *RUNX2* gene (NM_001024630.4)^7^ were investigated and compared with three aBMSCs from three unrelated healthy individuals. Cells were cultured in Dulbecco’s Modified Eagle Medium (DMEM, Gibco BRL, CA, USA) supplemented with 15% fetal bovine serum (HyClone, UT, USA), 1% L-glutamine, 100 U/ml penicillin, and 100 ug/ml streptomycin (Gibco BRL) in a 5% CO_2_ humidified atmosphere at 37 °C. The medium was changed twice a week. The p.Ser247Valfs*3 mutation in the aBMSCs was validated using Sanger sequencing with the primers shown in Table S3.

### Transfection and luciferase assay

HEK293 cells were transfected with 400 ng plasmids: pcDNA3.1[ +], WT-HA-RUNX2-pcDNA3.1[ +], p.Arg131Cys, p.Ser247Valfs*3, p.Pro279Ser, p.Asp287Asn, p.Gln301*, p.Gln361*, p.Glu366*, and p.Thr420Asn, 500 ng of the pOSE2-6Luc reporter plasmid (6 tandemly linked osteoblast-specific elements (OSE2) in the osteocalcin (OCN) promoter), and 100 ng Renilla expression plasmid (pRL-SV40, Promega, WI, USA) by lipofectamine 3000 (Invitrogen, CA, USA) according to the manufacturer’s instructions. Transfections were performed in three separate experiments, and each experiment was performed in triplicate. After 48 h, the cells were harvested and used in the dual-luciferase® reporter assay system (Promega).

### Western blot

Transfected HEK293 cells were lysed with 100 µl Pierce RIPA Buffer® (Thermo Scientific, Cleveland, OH, USA). The lysates were separated on a 12% SDS-PAGE gel and transferred to a polyvinylidene difluoride (PVDF) membrane. Mouse monoclonal anti-HA antibody (1:20,000, Sigma-Aldrich, Merck, MO, USA, Cat. No. H9658) was used as the primary antibody. The antibody against GAPDH (1:3000, Abcam, UK, Cat. No. ab8245) was used as a loading control. Mouse IgG HRP-conjugated Antibody (1:2500, R&D Systems, Inc, MN, USA, Cat. No. HAF007,) was used as a secondary antibody. Immunoblotting was performed using Thermo Scientific™ SuperSignal™ West Femto Maximum Sensitivity Substrate (Thermo Fisher Scientific, MS, USA) according to the manufacturer’s instructions.

### Flow cytometry analysis

The aBMSCs were characterized using flow cytometry. Expression of the surface markers CD44 (Biolegend®, CA, USA, Cat No. 338804), CD45 (Biolegend®, Cat No. 304026), CD73 (Biolegend®, Cat No. 344016), CD90 (Biolegend®, Cat No. 328118), and CD105 (Biolegend®, Cat No. 323206) were investigated.

### Osteogenic differentiation

The aBMSCs (2.5 × 10^4^ cells/cm^2^) were cultured in osteogenic medium containing growth medium supplemented with 250 nM dexamethasone (Sigma-Aldrich), 50 µg/mL ascorbic acid (Sigma-Aldrich), and 5 mM β-glycerophosphate (Sigma-Aldrich). Mineralization was examined using alizarin red S (ARS) staining after 7 days in osteogenic medium.

### Immunocytochemistry

The aBMSCs of the CCD patient, control cells, and transfected HEK293 cells were fixed with 4% paraformaldehyde and permeabilized with 0.1% Triton X-100 (Thermo Fisher Scientific). Non-specific binding was blocked with 1% bovine serum albumin. The cells were then incubated with the rabbit monoclonal anti-RUNX2 antibody (1:500, Abcam, Cambridge, UK, Cat. No. ab192256), followed by the secondary antibody, the donkey anti-rabbit IgG Alexa Fluor® 488-tagged (1:1000, Biolegend®, Cat. No. 406416), in combination with DAPI (1:2000, Roche, Cat. No. 10236276001) and Rhodamine Phalloidin Reagent (1:500, Abcam, Cat. No. ab235138). The cells were visualized using ApoTome.2 (Zeiss GmbH, Jena, Germany) at 100X magnification.

### Real‐time polymerase chain reaction (Real‐time PCR)

Total cellular RNA extracted from cells subjected to induced and noninduced osteogenic differentiation at day 7 was isolated using the RNeasy® Plus Mini Kit (Qiagen, Hilden, Germany). The concentration of the isolated RNA was measured using Thermo Scientific Nanodrop one (Thermo Scientific). The iScript Reverse Transcription (Bio‐rad Laboratories, Hercules, CA, USA) was used for converting RNA into cDNA. Realtime PCR was performed using the SYBR green detection system (FastStart Essential DNA Green Master, Roche Diagnostic) and the MiniOpticon real‐time PCR system (Bio‐Rad). The primer sequences are shown in Table S4.

### Statistical analysis

Data from the luciferase reporter assay (n = 9 for each variant) is presented as mean ± standard deviation (SD). The luciferase activity of the mutants and empty vector were compared with that of wildtype (WT) using the unpaired *t* test. The real-time PCR results of the mutant and three control aBMSCs were evaluated using the Mann‐Whitney *U* test (GraphPad Prism 8.0.2 Software Package, USA). Significance was defined as* P* value < 0.05 (*) and *P* value < 0.005 (**).

### Informed consent

The participant involved in the project consented for publication.

## Supplementary Information

Supplementary Information.

## Data Availability

All data generated or analyzed during this study are included in this published article and its Additional file 1.
