# Supplementary material for: Functional consequences of C-terminal mutations in RUNX2
Source: Sci Rep. 2023 Jul 27;13:12202. doi: 10.1038/s41598-023-39293-1 (PMC10374887; doi:10.1038/s41598-023-39293-1)
Supplement: Supplementary file 1 — Supplementary Information. [file 41598_2023_39293_MOESM1_ESM.pdf]

SUPPLEMENTARY DATA

Functional Consequences of C-Terminal Mutations in *RUNX2*

Sermporn Thaweesapphithak[1,2], Thanakorn Theerapanon[1], Khanti Rattanapornsompong[1], Narin Intarak[1], Pimsiri Kanpittaya[3], Vorapat Trachoo[4], Thantrira Porntaveetus[1,5*], Vorasuk Shotelersuk[6,7]

[1]Center of Excellence in Genomics and Precision Dentistry, Department of Physiology, Faculty of Dentistry, Chulalongkorn University, Bangkok, Thailand

[2]Graduate Program in Oral Biology, Faculty of Dentistry, Chulalongkorn University, Bangkok, Thailand.

[3]Department of Orthodontics, Faculty of Dentistry, Chulalongkorn University, Bangkok, Thailand

[4]Department of Oral and Maxillofacial Surgery, Faculty of Dentistry, Chulalongkorn University, Bangkok, Thailand

[5]Graduate Program in Geriatric and Special Patients Care, Faculty of Dentistry, Chulalongkorn University, Bangkok, Thailand.

[6]Center of Excellence for Medical Genomics, Medical Genomics Cluster, Department of Pediatrics, Faculty of Medicine, Chulalongkorn University, Bangkok, Thailand

[7]Excellence Center for Genomics and Precision Medicine, King Chulalongkorn Memorial Hospital, the Thai Red Cross Society, Bangkok, Thailand

	Arg131	Pro279 … Asp287	Thr420	
Homo sapiens	SHWRCNK	PFNPQGQSQITDPRQ	HYHTYLP	**NP_001019801.3**
Mus musculus	SHWRCNK	PFNPQGQSQITDPRQ	---TVPA	**XP_006523603.1**
Danio rerio	SHWRCNK	SFNPQGQTQISDPRQ	HYHTYLP	**NP_998023.1**
Rattus norvegicus	SHWRCNK	PFNPQGQSQITDPRQ	HYHTYLP	**XP_006244611.1**
Caenorhabditis elegans	KHWRSNK	SF-------------	IFIT---	**NP_491679.1**
Xenopus tropicalis	SHWRCNK	PFNPQGQSQIADPRQ	HYHTYLP	**NP_001128588.1**
Drosophila melanogaster	NHWRSNK	AGQP--AAMMPSPPG	-------	**NP_001285501.1**

Fig S1. Amino acid conservations of p.Arg131, Pro279, Asp287, and Thr420 among several species.

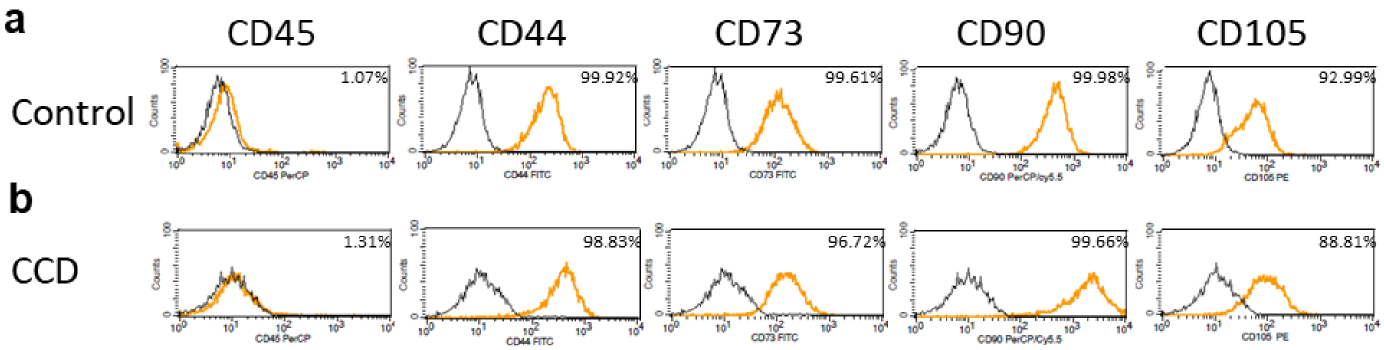

Fig S2. Flow cytometric analysis of aBMSCs marker expression. a, b. CCD and control cells were positive for CD44, CD73, CD90, and CD105, but negative for CD45.

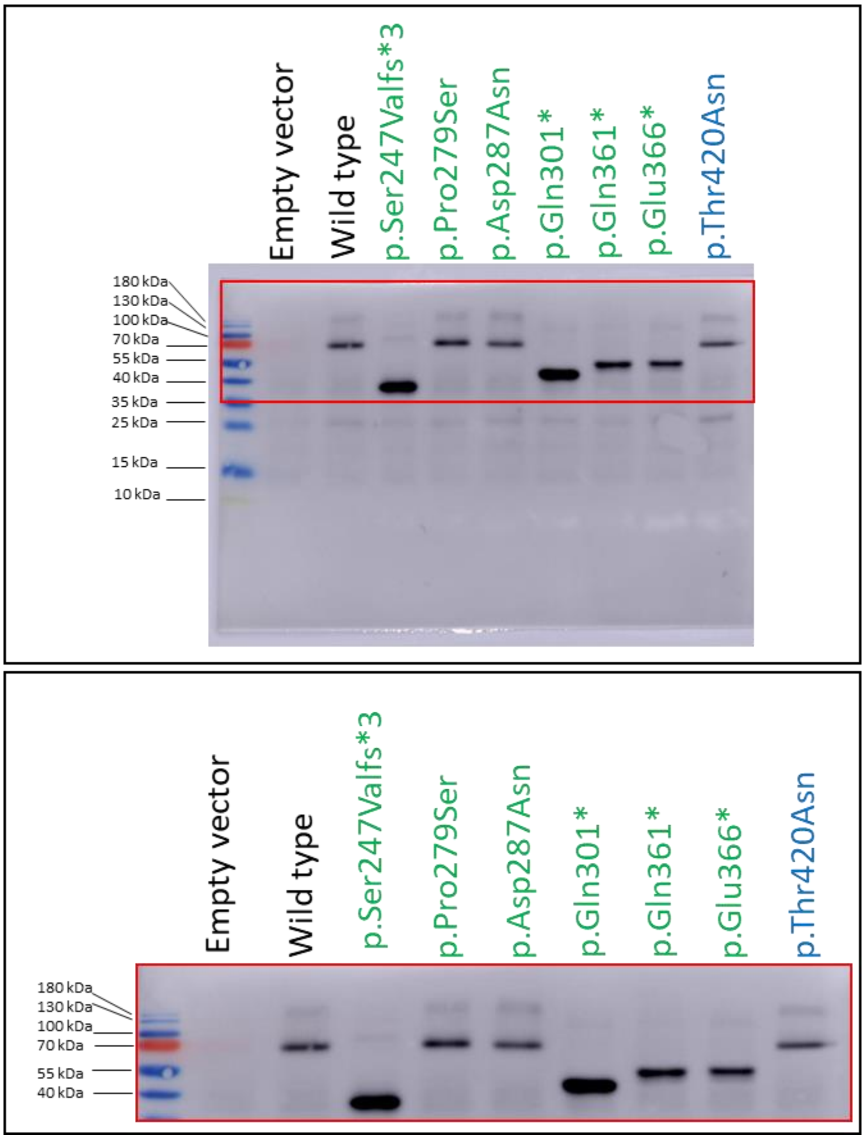

Fig S3. Full length blot of RUNX2 expression in empty, wildtype, and mutants (p.Ser247Valfs*3, p.Pro279Ser, p.Asp287Asn, p.Gln301*, p.Gln361*, p.Glu366*, and p.Thr420Asn) vectors transfected in HEK293 cells. Red boxes indicate the cropped blot shown in Figure 1c.

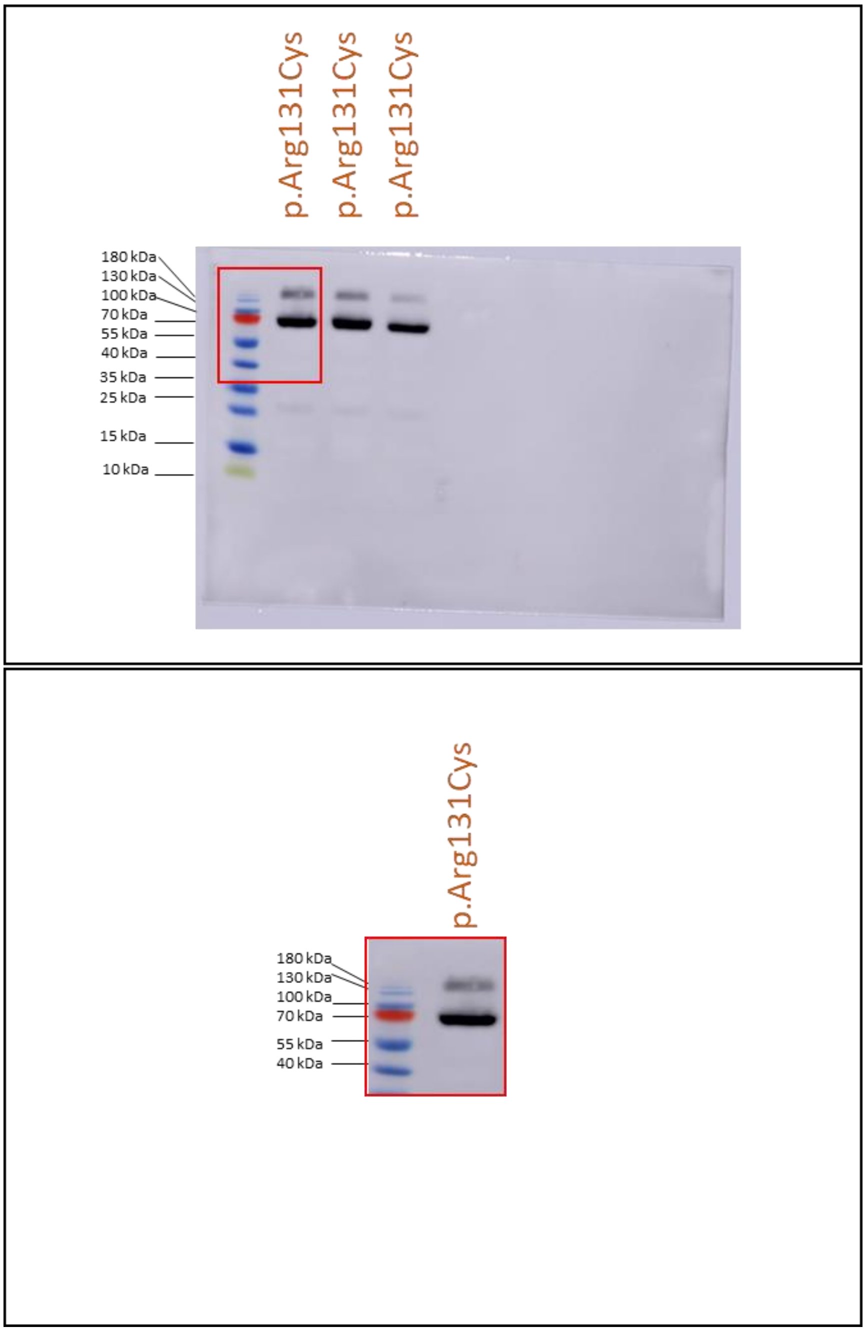

Fig S4. Full length blot of RUNX2 expression in mutants (p.Arg131Cys) vectors transfected in HEK293 cells. Red boxes indicate the cropped blot shown in Figure 1c.

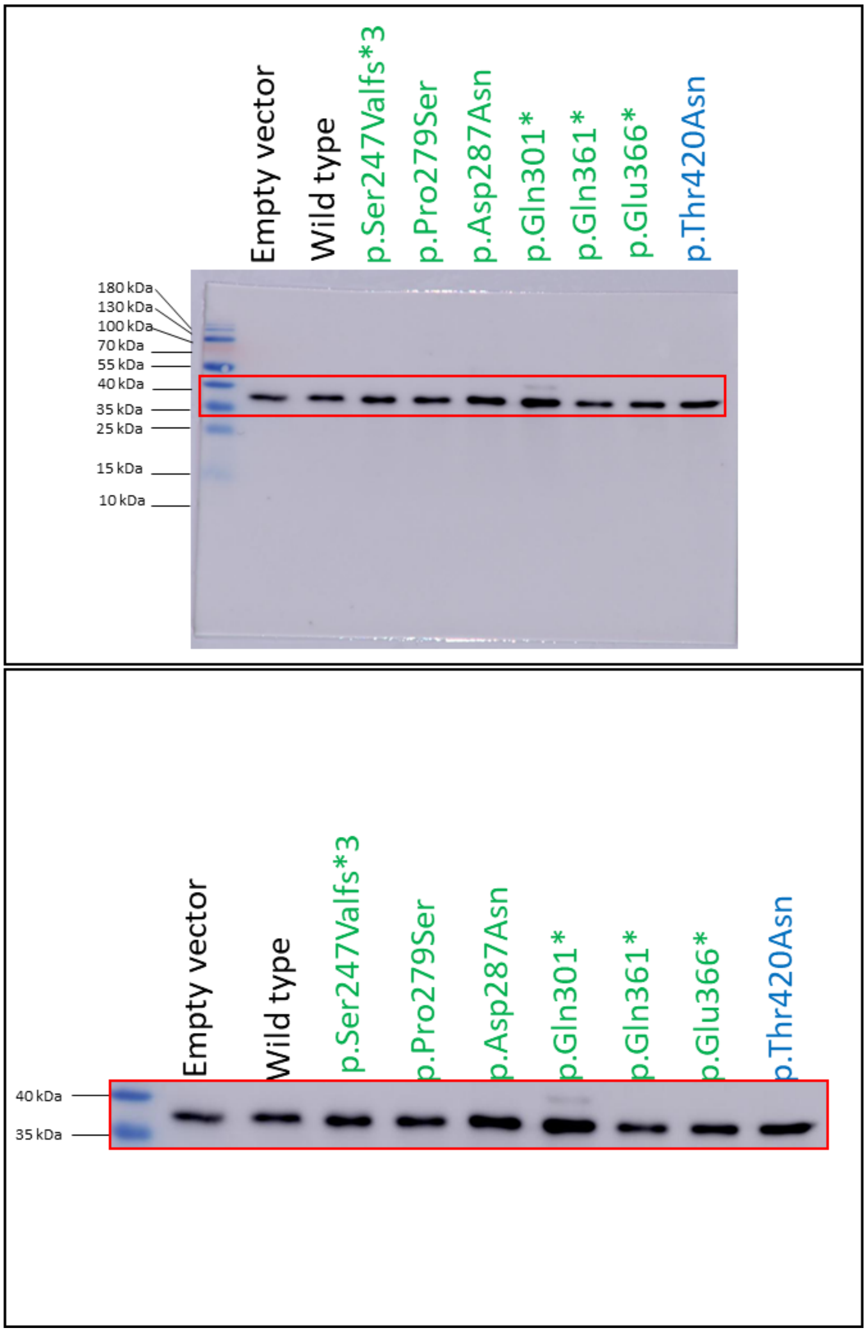

Fig S5. Full length blot of GAPDH expression in empty, wildtype, and mutants (p.Ser247Valfs*3, p.Pro279Ser, p.Asp287Asn, p.Gln301*, p.Gln361*, p.Glu366*, and p.Thr420Asn) vectors transfected in HEK293 cells. Red boxes indicate the cropped blot shown in Figure 1c.

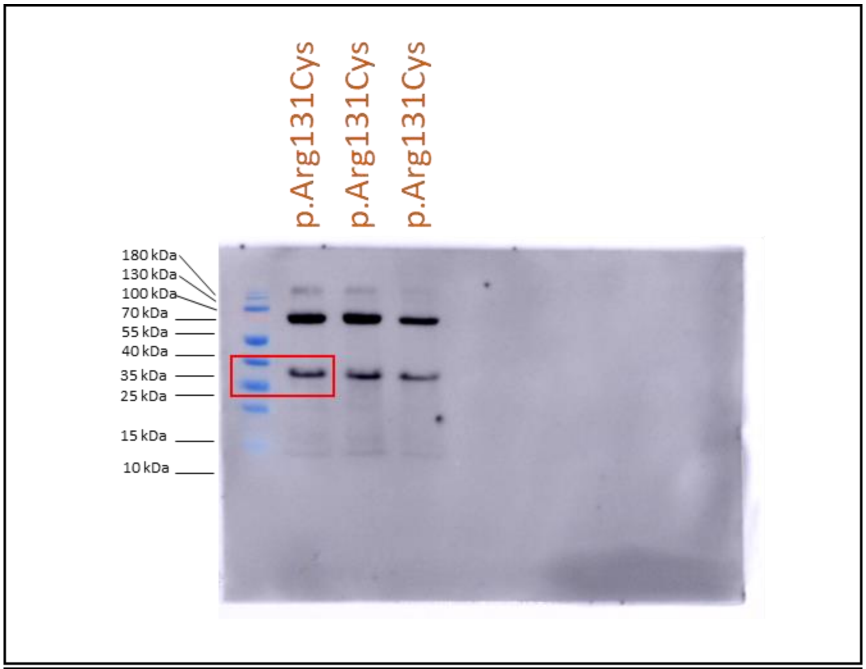

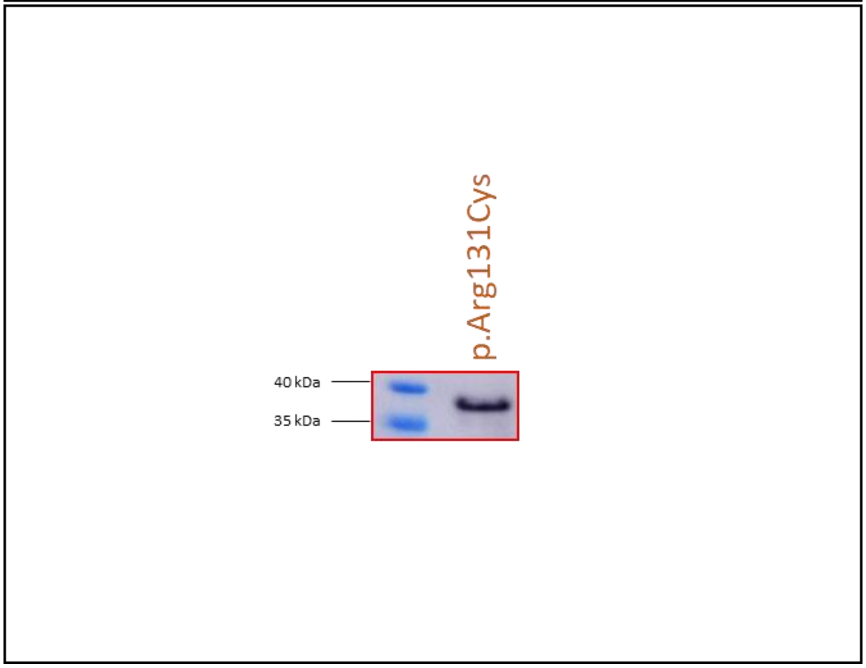

Fig S6. Full length blot of GAPDH expression in mutants (p.Arg131Cys) vectors transfected in HEK293 cells. Red boxes indicate the cropped blot shown in Figure 1c.

Table S1. Primers used for site-directed mutagenesis.

Variants	Primers	Sequence 5'> 3'	Annealing temperature [°C]
c.A739del<br>p.Ser247Valfs*3	Forward<br>Reward	TCTCTGACCGCCTCGTGATTTAGGGC<br>GC GAGGCGGTCAGAGAACAA	60.6
c.835C>T<br>p.Pro279Ser	Forward<br>Reward	TCCTTTTAATTCACAAGGACAGAG<br>CTTGGTGCAGAGTTCAGG	53.0
c.859G>A<br>p.Asp287Asn	Forward<br>Reward	TCAGATTACAAACCCCAGGCA<br>CTCTGTCCTTGTGGATTAAAAGG	54.9
c.901C>T<br>p.Gln301*	Forward<br>Reward	GTCCTATGACTAGTCTTACCCCTCC<br>CACGGCGGGGAAGACTGT	58.5
c.1081C>T<br>p.Gln361*	Forward<br>Reward	TAAGAAGAGCTAGGCAGGTGC<br>CTGAGAGTGGAAGGCCAG	56.0
c.1096 G>T<br>p.Glu366*	Forward<br>Reward	AGGTGCTTCATAACTGGGCCC<br>GCCTGGCTCTTCTTACTG	55.5
c.1259C>A<br>p.Thr420Asn	Forward<br>Reward	CACTACCACAACTACCTGCCA<br>AGTGGTGGCGGACATACC	57.0

Table S2. Primers used for Sanger sequencing to validate the mutagenesis.

Primer	Sequence 5'> 3'	Annealing temperature [°C]
Forward<br>Reward	GCGCATTCCTCATCCCAGTA<br>GGCTCAGGTAGGAGGGGTAA	60

Table S3. Primers used for Sanger sequencing of the alveolar bone mesenchymal stem cells (aBMSC) obtained from the CCD patient harboring the p.Ser247Valfs*3 mutation.

Primer	Sequence 5'> 3'	Annealing temperature [°C]
Forward	TGGCCACCAGATACCGCTTA	60
Reward	ATAAGCCGCTTCACAGCTCC	

Table S4. Primers used for real-time PCR.

Genes	Primers	Primer Sequence (5' > 3')	Annealing temperature [°C]
ALP	Forward	CGA GAT ACA AGC ACT CCC ACT TC	60
	Reverse	CTG TTC AGC TCG TAC TGC ATG TC	
COL1A1	Forward	CTG GCA AAG AAG GCG GCA AA	60
	Reverse	CTC ACC ACG ATC ACC ACT CT	
OCN	Forward	CTT TGT GTC CAA GCA GGA GG	60
	Reverse	CTG AAA GCC GAT GTG GTC AG	
RUNX2	Forward	ATG ATG ACA CTG CCA CCT CTG A	60
	Reverse	ATG GAG GGC GGA TTG GAA A	
β-actin	Forward	TGG AAC GGT GAA GGT GAC AG	60
	Reverse	AAC AAC GCA TCT CAT ATT TGG AA	
